# Supplementary material for: The Role of the Serotonergic System in Time Perception: A Systematic Review
Source: Int J Mol Sci. 2024 Dec 11;25(24):13305. doi: 10.3390/ijms252413305 (PMC11679555; doi:10.3390/ijms252413305)
Supplement: Supplementary file 1 [file ijms-25-13305-s001.zip › ijms-3339549-supplementary.pdf]

S1. Modifications into the OHAT risk of bias tool made for the human studies assessment. Generally, the risk of bias of the included studies was assessed according to the OHAT risk of bias tool and using the following scale of marks for each question: "++" – definitely low risk of bias; "+" – probably low risk of bias; "–" – probably high risk of bias, "– –" – definitely high risk of bias. For the total score calculation the OHAT tool marks were translated to values in the following way: "++" – 3 points, "+" – 2 points, "–" – 1 point, "– –" – 0 points.

The selection bias section. The questions of this section were merged into one assessing adequacy of the randomization and group allocation for the studies with substance administration and whether the comparison groups were appropriate for studies with genetic analysis of interindividual differences and with clinical samples.

Studies were marked with "++" in case of proper randomization in studies with substance administration or proper comparison groups in terms of general characteristics, such as age, gender, etc. Studies were marked with "+" in case of mild faults.

The confounding and performance bias sections stayed unchanged.

In the confounding bias section, studies were marked with "+" in case of consideration and assessment of possible confounders (in none of the studies confounders were adjusted in the statistical analysis). Studies were marked with "–" when the confounders were not considered. In the performance bias section, studies were marked with "++" in case of reported double-blind design; with "+" – in case of unreported but logically inferred blindness; with "–" – in case of reported single-blind design or absent blindness of the researches to the clinical groups.

The detection bias section. We modified the question: "Can we be confident in the exposure characterization?" to "Can we be confident in the reliability of the serotonergic function assessment?". In order to evaluate the strength of evidence in relation to assessment of serotonergic neurotransmission. Studies using direct influences on serotonergic system were given "++"; studies using indirect inferences or providing only correlational evidence were marked with "+".

We modified the question: "Can we be confident in the outcome assessment?" to "Can we be confident in Time Perception assessment?". In order to evaluate the reliability of the experimental paradigm used to assess time perception. Studies using reliable psychophysical procedures with multiple trials were marked with "++"; studies using less reliable methods were marked with "+"; a study using only one unreliable measure with only one trial was marked with "– –".

Other sources of bias section was focused on the appropriateness of the statistical methods used. Studies using reliable statistical methods with no visible faults were marked with "++", with mild inaccuracy – with "+"; studies with an obvious fault (e.g. proceeding to post-hoc analysis after a statistical tendency) were marked with "–"; studies with pronounced inaccuracies were marked with "– –".

The attrition/exclusion and selective reporting bias sections (and corresponding questions) were excluded because this category is irrelevant for the studies included.

Table S1. Quality assessment of the human studies included.

	Selection bias	Confounding bias	Performance bias	Detection bias		Other sources of bias	
	1. Can we be confident in randomisation/ group allocation or whether the comparison groups were appropriate?	2. Did the study design or analysis account for important confounding and modifying variables?	3. Were the research personnel and human subjects blinded to the study group during the study?	4. Can we be confident in the reliability of the serotonergic function assessment?	5. Can we be confident in Time Perception assessment?	6. Were the statistical methods used appropriate?	Total score
Leigh et al., 1991	3	1	1	3	0	0	8
Wittmann et al., 2007	3	2	3	3	3	3	17
Portnova et al., 2007	2	1	2	2	2	2	11
Tanaka et al., 2007	3	2	3	2	3	3	16
Schweighofer et al., 2008	3	2	3	2	3	3	16
Wackerman et al., 2008	3	2	3	3	3	3	17
Sysoeva et al., 2010	3	2	2	2	3	3	15
Yanakieva et al., 2019	3	1	3	3	2	3	15
Mavrogiorgou et al., 2022	2	2	1	2	2	1	10
Medvedeva et al., 2023	2	2	1	2	3	3	13

S2. Modifications into the OHAT risk of bias tool made for the animal studies assessment. Generally, the risk of bias of the included studies was assessed according to the OHAT risk of bias tool and using the following scale of marks for each question: "++" – definitely low risk of bias; "+" – probably low risk of bias; "–" – probably high risk of bias, "– –" – definitely high risk of bias. For the total score calculation the OHAT tool marks were translated to values in the following way: "++" – 3 points, "+" – 2 points, "–" – 1 point, "– –" – 0 points.

The assessment questions were used as originally proposed by the OHAT Risk of Bias Tool.
Randomisation. Was the administered dose or exposure level adequately randomized?
Allocation concealment. Was allocation to study groups adequately concealed?
Identical experimental conditions. Were experimental conditions identical across study groups?
Blinding during the research. Were the research personnel and human subjects blinded to the study group during the study?
Missing outcome data. Were outcome data complete without attrition or exclusion from analysis?
Exposure characterization. Can we be confident in the exposure characterization?
Outcome assessment. Can we be confident in the outcome assessment?
Outcome reporting. Were all measured outcomes reported?
Confounding (Design/Analysis). Were there no other potential threats to internal validity (e.g., statistical methods were appropriate and researchers adhered to the study protocol)?

Table S2. Quality assessment of the animal studies included.

	Randomization	Allocation concealment	Identical experimental conditions	Blinding during the research	Missing outcome data	Exposure characterization	Outcome assessment	Outcome reporting	Confounding (Design/ Analysis)	Summary, of 27
Wogar et al. 1992	1	1	3	1	2	2	2	3	2	17
Wogar et al. 1993	1	1	3	1	2	2	2	3	2	17
Sokolowski& Seiden 1999	2	1	2	1	3	2	2	2	2	17
Sabol et al. 2000	1	0	2	1	1	2	2	3	2	14

Scott-McKean et al. 2009	2	1	2	1	2	3	3	2	2	18
Malikowska-Racia et al. 2022	2	1	2	1	2	2	2	3	2	17
Malikowska-Racia et al. 2023	2	1	2	1	2	3	2	2	3	18
AI-Zahrani et al. 1996	1	1	3	1	2	2	2	2	2	16
Bizot et al. 1997	1	1	2	1	2	3	2	2	3	17
Chiang et al. 1999	2	1	2	1	2	2	2	2	2	16
Chiang et al. 2000	2	1	2	1	2	2	2	2	2	16
Body et al. 2001	2	1	3	1	2	2	2	3	2	18
Body et al. 2002	1	1	3	1	2	2	2	1	2	15
Body et al. 2003	3	1	3	1	3	2	2	1	2	18
Body et al. 2005	3	1	3	1	2	2	2	2	2	18
Body et al. 2006a	2	1	2	2	2	2	2	2	2	17
Body et al. 2006b	2	1	2	1	2	2	2	2	2	16
Cheung et al. 2007	1	1	3	1	2	2	2	2	2	16
Body et al. 2009	1	1	3	1	2	2	2	2	2	16
Body et al. 2014	1	1	2	1	2	3	2	2	2	16

Morrissey et al. 1993	2	1	3	1	2	2	2	2	2	17
Ho et al. 1995	3	1	3	1	2	2	2	2	2	18
Al-Zahrani et al. 1996	2	1	3	1	2	2	2	2	2	17
Body et al. 2002	1	1	3	1	2	2	2	2	2	16
Asgari et al. 2005	3	1	3	1	2	2	2	2	2	18
Asgari et al. 2006	3	1	3	1	2	2	2	2	2	18
Hampson et al. 2010	3	1	2	1	2	2	2	2	2	17
Popik et al. 2022	1	1	3	1	3	3	2	2	3	19
Avlar et al. 2015	3	1	3	1	2	2	2	1	1	16
Halberstadt et al. 2016	3	1	3	1	2	2	2	2	2	18
Morrissey et al. 1994	1	1	3	1	2	2	2	2	2	16
Ho et al. 1996	2	1	2	1	2	2	2	2	2	16
Bayley et al. 1998	2	1	1	1	2	2	1	2	1	13
MacDonald, Meck 2005	2	1	1	1	2	2	1	2	1	13
Asgari et al. 2006	2	1	2	1	2	2	2	2	2	16

Buhusi and Meck 2007	2	1	1	1	2	3	1	1	1	13
Heilbronner, Meck 2014	2	1	1	1	2	2	1	2	1	13
Shapiro et al. 2018	2	1	1	1	2	2	1	1	2	13
Dellu-Hagedorn et al. 2018	2	2	2	1	1	2	3	2	2	17
Acosta et al. 2018	2	1	2	1	2	3	3	2	2	18
Miyazaki et al 2020	1	1	2	2	2	3	3	3	3	20
Matsunami et al. 2012	2	2	1	0	1	2	3	2	2	15
Zaichenko et al. 2013	2	1	3	1	2	2	2	2	2	17
Zaichenko et al. 2014	2	1	3	1	2	2	2	2	2	17
Kirkpatrick et al. 2014	2	1	0	1	2	2	1	2	2	13
Buhusi et al. 2017	1	1	1	1	2	3	3	2	3	17
Jiang et al. 2022	1	1	2	1	2	2	2	3	3	17
Roberts et al. 2023	1	1	2	2	2	2	2	3	2	17

Inaba et al. 2013	0	1	3	1	2	3	2	2	2	16
McLaughlin et al. 2017	1	2	2	1	2	2	2	2	2	16
